# Primary aldosteronism caused by a pI157S somatic *KCNJ5* mutation in a black adolescent female with aldosterone-producing adenoma

**DOI:** 10.3389/fendo.2022.921449

**Published:** 2022-08-16

**Authors:** Celso E. Gomez-Sanchez, Desmaré van Rooyen, William E. Rainey, Kazutaka Nanba, Amy R. Blinder, Radhakrishna Baliga

**Affiliations:** ^1^ Endocrine Section, G.V. Sonny Montgomery VA Medical Center, Jackson, MS, United States; ^2^ Department of Pharmacology and Toxicology, University of Mississippi Medical Center, Jackson, MS, United States; ^3^ Department of Molecular and Integrative Physiology, University of Michigan, Ann Arbor, MI, United States; ^4^ Division of Metabolism, Endocrinology, and Diabetes, Department of Internal Medicine, University of Michigan, Ann Arbor, MI, United States; ^5^ Department of Endocrinology and Metabolism, National Hospital Organization Kyoto Medical Center, Kyoto, Japan; ^6^ Division of Nephrology, Department of Pediatrics, Louisiana State University, Shreveport, LA, United States

**Keywords:** primary aldosteronism, KCNJ5 mutation, aldosterone, pediatric hypertension, adrenal adenoma

## Abstract

Aldosterone-producing adenoma is a rare cause of hypertension in children. Only a limited number of cases of aldosterone-producing adenomas with somatic *KCNJ5* gene mutations have been described in children. Blacks are particularly more susceptible to developing long-standing cardiovascular effects of aldosterone-induced severe hypertension. Somatic *CACNA1D* gene mutations are particularly more prevalent in black males whereas *KCNJ5* gene mutations are most frequently present in black females. We present here a novel somatic *KCNJ5* p.I157S mutation in an aldosterone-producing adenoma from a 16-year-old black female whose severe drug-resistant hypertension significantly improved following unilateral adrenalectomy. Prompt diagnosis of aldosterone-producing adenoma and early identification of gene mutation would enable appropriate therapy and significantly reduce cardiovascular sequelae.

## Introduction

Primary aldosteronism (PA) is the most common secondary cause of hypertension (HTN) with a prevalence between 5-22% of adult hypertensive patients (1, 2). Two forms of PA are the most common presentations: namely aldosterone-producing adenomas (APA) accounting for 30-70% of patients and bilateral zona idiopathic hyperaldosteronism (IAH also accounting for 30-70% of patients with rare cases of unilateral hyperplasia, aldosterone-producing carcinomas and familial hyperaldosteronism accounting for 1-2% (1). Sporadic cases of PA are rare in the pediatric population (3–5). Only a limited number of cases have been reported in the pediatric literature, most of which have been of the familial type 3 (5–7) or presenting with a mosaicism (8) all bearing a mutation of the potassium voltage-gated channel subfamily J member 5 gene (*KCNJ5*). A somatic mutation of the *KCNJ5* gene mutated at p.L168R was first described in a child with moderate to severe hypertension and hypokalemia and low renin with high aldosterone levels by Uchida et al. (3). In addition to mutations of the KCNJ5 channel, calcium (*CACNA1D* and *CACNA1H*) and chloride (*CLCN2*) channel gene mutations have been reported in adults and in familial cases and somatic mutations of the sodium ATPase pump (*ATP1A1*) and calcium pump (*ATP2A3*) mutations in adults have been described (9–12). Using a combination of CYP11B2 immunohistochemistry-guided DNA capture with sequencing has resulted in the identification of somatic mutations in approximately 94% of APAs (13). There is a racial and sex difference in the prevalence of the different mutations with *KCNJ5* being more common in East Asians and women (10–13). We report here a novel somatic *KCNJ5* mutation in a 16-year-old black female with APA whose HTN significantly improved following unilateral adrenalectomy.

## Case report

**Figure 1:**
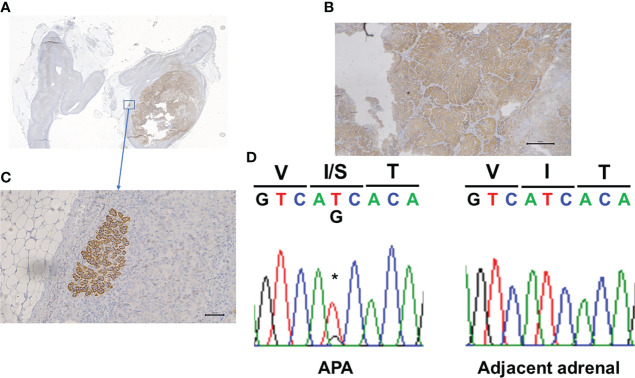
Panel **(A, B)** shows CYP11B2 immunohistochemistry demonstrating relative homogenous expression within the adrenal adenoma. Panel **(C)** shows the presence of an Aldosterone-Producing Cell Clusters. Panel **(D)** shows the Sanger demonstration of the mutation of nucleotide. *Site of the mutation.

16-year-old black women was noted to be hypertensive while being evaluated for depression. She was referred a year later for recurrent headaches, chronic drug resistant hypertension and persistent hypokalemia. Her therapeutic regimen consisted in lisinopril 40mg daily, amlodipine 10 mg daily, amiloride 15 mg daily, potassium chloride 40 mEq twice daily, labetalol 500 mg three times per day and minoxidil 5 mg. Family history was positive for a maternal uncle with hypertension. On examination, her weight was 98 kg [> 99%], height 178 cm [94%], BMI 36.00kg/m^2^, heart rate 88 beats per minute and blood pressure (BP) 155/94 mmHg [> 95%]. Pertinent laboratories: serum sodium 142, potassium 2.8, chloride 106, and CO_2_ content 26 mEq/L. Serum creatinine was 0.80 (estimated GFR 100 mL/mt/1.73m^2^), and BUN 10 mg/dL. Plasma aldosterone concentration (PAC) was 27.2 ng/dL and plasma renin activity (PRA) <0.6 ng/mL/h with PAC/PRA ratio of >45 [significant > 20]. Cortisol level was 8.7 [normal range (N) 1.7-14.1] μg/dl. Timed urine aldosterone for estimated urine creatinine of 1980 mg was 20 µg/d [N <12]. Liquid chromatography-tandem mass spectrometry (LC-MS/MS) quantification of 29 steroids in the pre-operatory serum was done (14). Serum levels of 18-oxocortisol and 18-hydroxycortisol were clearly elevated (**Table 1**) (15). Cardiac echocardiogram showed compaction cardiomyopathy. CT abdomen indicated a 1.3 x 2.7 cm right adrenal nodule measuring -16 Hounsfield units suggesting a benign adrenal adenoma. The contralateral adrenal had normal imagen characteristics. At the time of surgery her serum creatinine was 1.0 mg/dL (estimated GFR of 80 mL/mt/1.73m^2^). Written informed consent was obtained for surgery and pathological and genome studies. Robotic right adrenalectomy was performed, and pathology was consistent with an adrenocortical adenoma. Immunohistochemistry using a CYP11B2 monoclonal antibody (16) showed diffuse staining of the adenoma (APA) (**Figures 1A, B**) and the presence of at least one aldosterone-producing micronodule (**Figure 1C**). Genomic DNA samples from formalin-fixed paraffin embedded right adrenal tissue were sent for sequencing for germline or somatic mutations. CYP11B2 (aldosterone synthase) immunohistochemistry-guided targeted next-generation sequencing (NGS) of APA (11) revealed a somatic mutation in the *KCNJ5* gene (nucleotide change c.T470G resulting in amino acid change p.I157S), with a variant allele frequency of 33%. The adjacent adrenal did not have the mutation. The results were confirmed by Sanger sequencing (**Figure 1D**) (11). Post-operatively PAC was < 3.0 ng/dL. Her headaches resolved; her blood pressure significantly improved to 124/69 mmHg [<90%] with normalization of her serum potassium. Six months after her right adrenalectomy BP was 108/63 mm Hg on a single antihypertensive agent and her serum potassium levels remain normal. Her repeat echocardiogram showed no significant change except for slight improvement in the left ventricular ejection fraction.

**Table 1:** LC-MS/MS quantification of 29 steroids in serum.

	Patient levels			Eisenhofer “Normal”
			Follicular Phase	Luteal Phase
	ng/dL	nmol/L	nmol/L (median + ranges)	nmol/L (median + ranges)
**Preg-S**	4212.53	106.24		
**17OHPreg-S**	539.56	13.08		
**DHEA-S**	68168.91	1849.90	3741 [1186–7728]	4108 [1156–8217]
**A5-S**	3407.34	91.97		
**Prog**	500.78	15.92	0.56 [0.06–18.64]	13.23 [0.07–83.00]
**DOC**	14.81	0.45	0.19 [0.01–0.50]	0.27 [0.01–0.57]
**CORT**	221.90	6.40	5.76 [2.0–87.2]	6.26 [1.88–36.1]
**18OH-CORT**	81.66	2.25		
**ALDO**	19.10	0.53	0.17 [0.02–0.65]	0.19 [0.03–0.88]
**17OHProg**	133.85	4.05	1.08 [0.36–4.99]	2.99 [0.37–8.28]
**16OHProg**	38.44	1.16		
**11-deoxycortisol**	80.76	2.33	0.40 [0.12–1.52]	0.45 [0.15–3.81]
**Cortisol**	8679.84	239.47	297 [97–979]	295 [150–822]
**Cortisone**	1630.22	45.23	55.1 [28.9–87.9]	63.2 [34.4–92.1]
**18OH-Cortisol**	402.58	10.64	1.71 [0.36–5.15]	1.68 [0.61–3.33]
**18oxo-Cortisol**	91.67	2.44	0.03 [0.00–0.09]	0.03 [0.00–0.09]
**11OHProg**	ND	ND	–	–
**11KProg**	ND	ND	–	–
**21dF**	ND	ND	0.04 [0.00–0.22]	0.02 [0.00–0.54]
**A4**	79.34	2.77	3.10 [1.80–6.91]	4.19 [1.26–12.81]
**11OHA4**	74.01	2.45	–	–
**11KA4**	18.87	0.63	–	–
**T**	17.18	0.60	0.93 [0.42–1.92]	1.13 [0.27–2.18]
**11OHT**	ND	ND	–	–
**11KT**	16.20	0.54	–	–
**DHT**	14.46	0.50	–	–
**Estrone**	ND	ND	–	–
**Estradiol**	ND	ND	–	–
**Estriol**	ND	ND	–	–

ND, not detected.

Preg-S, pregnenolone sulfate; DHEA-S, dehydroepiandrosterone sulfate; A5-S, 5-androstene, 3β, 17β diol-3 sulfate; Prog, progesterone; DOC, deoxycorticosterone; CORT, corticosterone; 18OH-CORT, 18-hydroxycorticosterone; ALDO, aldosterone; 17OHProg,17α-hydroxyprogesterone; 16PHProg, 16α-hydroxyprogesterone; 18OH-Cortisol, 18-hydroxycortisol; 11OHProg, 11β-hydroxyprogesterone; 11KProg, 11-ketoprogesterone; 21dF, 21-deoxycortisol; A4, androstenedione; 11OHA4- 11β-hydroxyandrostenedione; 11KA4, 11-keto-androstenedione; T, testosterone; 11OHT, 11β-hydroxytestosterone; DHT, 5α-dihydrotestosterone.

## Discussion

HTN in the black population continues to be a significant cardiovascular (CV) risk factor with an overall increase in morbidity and mortality that has led to race specific clinical guidelines (17). African Americans appear to be more susceptible to the blood pressure effects of aldosterone (17). The prevalence of PA in African Americans in comparison to white individuals is not known. The pattern of somatic mutations in APA is different in the various ethnic groups and between sexes (10–13, 18). *KCNJ5* mutations are more prevalent in East Asians, American and European white males and in females, including African Americans (10–13, 18), while *CACNA1D* mutations appear to be more prevalent in African American males (11).

Mutation in the *KCNJ5* gene results in increased intracellular calcium concentration which activates the transcription of the aldosterone synthase gene *CYP11B2* leading to autonomous aldosterone production (7, 19). Targeting the entire coding region for sequencing of genes mutated in APAs based on the tumor expression of CYP11B2, required for the final steps of aldosterone production, provides more accurate determination of the APA-related somatic mutations than the conventional mutation hot spot sequencing (10–12). Utilizing this technique, we sequenced DNA from the CYP11B2 positive tumor and CYP11B2 negative adjacent normal tissue and demonstrated the presence of somatic *KCNJ5* mutation p.I157S in the tumor and not the adjacent normal area. This mutation has been previously characterized and reported only as a germline mutation in two patients from a single family. Both patients had severe primary hyperaldosteronism, early refractory HTN and bilateral massive adrenal hyperplasia (20) A single heterozygous thymine to guanine (T to G) substitution was noted at nucleotide position 470 (c.T470G) which resulted in isoleucine (I) to serine (S) substitution at the amino acid 157 (p.I157S). This mutation close to the channel pore has been shown to result in loss of potassium selectivity, cell membrane depolarization, increased Ca^2^ entry in adrenal glomerulosa cells and increased aldosterone production (20). A patient with uncontrolled bilateral aldosteronism treated with bilateral adrenalectomy was found to have 11 different adrenal nodules all of which had a disease-causing mutation in the *KCNJ5* gene (p.G151R) and no family history of the mutation (8). Deep sequencing showed that 0.23% of germline DNA carried the same variant as the adrenal nodules (8) demonstrating the presence of a low grade mosaicism that manifested only in the adrenal nodules. We did not do deep sequencing but this is unlikely in this patient that had a single unilateral adenoma.

Kidney damage in APA can occur initially with the aldosterone-induced expansion of the intravascular fluid volume which can result in glomerular hyperfiltration. It can then result in intra glomerular hypertension leading to glomerular damage, albuminuria, and compromised kidney function. Our patient had prolonged persistent severe resistant HTN resulting in compromised kidney function that significantly improved following unilateral adrenalectomy. Mineralocorticoid receptor antagonist (MRA) has been shown to be reno-protective but was not initiated until prior to surgery due to the delay in diagnosis of her APA.

The outcome of patients following adrenalectomy for unilateral PA is found to be better in younger patients who were more likely to show complete clinical recovery when compared to older patients. Women as a whole have a greater chance of complete clinical success than men, however complete or partial success was limited in the presence of higher BMI and prior long standing untoward effects of vascular and renal changes resulting from long standing aldosterone induced HTN (21). In a study of Asian population with APA following unilateral adrenalectomy Nishikawa et al. (22) observed that those with a *KCNJ5* mutation taking fewer anti hypertensives and with shorter duration of HTN had complete resolution of HTN with significant improvement in their LV hypertrophy when compared to the wild type. Our patient over a six-month period following unilateral adrenalectomy had significant improvement in her HTN requiring only Lisinopril. Her kidney function has also improved significantly being close to her baseline. However, her compaction cardiomyopathy and her LV ejection fraction continues to remain unchanged. Her BMI continues to remain high with her being black potentially contributing to the substantial delay in her successful clinical improvement.

In conclusion unilateral APA should be considered in any child presenting with drug resistant HTN with or without hypokalemia in order to ensure early diagnosis and adrenalectomy that would prevent significant CV sequelae. The identification of somatic and germline mutations will further provide insight into the mechanism of APA and assist in tailoring appropriate therapy particularly in blacks who appear to have higher CV disease morbidity and mortality resulting from autonomous aldosterone secretion.

## Data availability statement

The datasets for this article are not publicly available due to concerns regarding participant/patient anonymity. Requests to access the datasets should be directed to the corresponding author.

## Ethics statement

Ethical review and approval was not required for the study on human participants in accordance with the local legislation and institutional requirements. Written informed consent to participate in this study was provided by the participants’ legal guardian/next of kin.

## Author contributions

CG-S conceived the study, performed experiments and wrote the paper. DR performed measurements, edited the paper, KN performed and interpreted the sequencing, edited the paper. AB performed and interpreted the sequenes and edited the paper, WR directed some of the experiments, edited the paper and RB studied the patient, wrote the initial paper and edited the final copy. All authors contributed to the article and approved the submitted version.

## Funding

Research reported in this publication was supported by National Heart, Lung and Blood Institute grant R01 HL144847 (CEGS), the National Institute of General Medical Sciences grant U54 GM115428 (CEGS), Department of Veteran Affairs BX00468 (CEGS), the National Institute of Diabetes and Digestive and Kidney grant R01 DK43140 (WER), and a Japan Heart Foundation Grant (KN). The content is solely the responsibility of the authors and does not necessarily represent the official views of the National Institutes of Health.

## Acknowledgments

The authors would like to thank Dr. Aaron M. Udager and Chia-Jen Liu at the University of Michigan for next-generation sequencing.

## Conflict of interest

The authors declare that the research was conducted in the absence of any commercial or financial relationships that could be construed as a potential conflict of interest.

## Publisher’s note

All claims expressed in this article are solely those of the authors and do not necessarily represent those of their affiliated organizations, or those of the publisher, the editors and the reviewers. Any product that may be evaluated in this article, or claim that may be made by its manufacturer, is not guaranteed or endorsed by the publisher.
